# Phylogenetic relationships of subfamilies in the family Hesperiidae (Lepidoptera: Hesperioidea) from China

**DOI:** 10.1038/srep11140

**Published:** 2015-06-10

**Authors:** Xiangqun Yuan, Ke Gao, Feng Yuan, Ping Wang, Yalin Zhang

**Affiliations:** 1 Key Laboratory of Plant Protection Resources and Pest Management of Ministry of Education, Entomological Museum of Northwest A&F University, 712100, Yangling, Shaanxi, China; 2 Department of Entomology, Cornell University, 14456, Geneva, NY, USA

## Abstract

Hesperiidae is one of the largest families of butterflies. Our knowledge of the higher systematics on hesperiids from China is still very limited. We infer the phylogenetic relationships of the subfamilies of Chinese skippers based on three mitochondrial genes (cytochrome b (*Cytb*), the NADH dehydrogenase subunit 1 (*ND1*) and cytochrome oxidase I (*COI*)). In this study, 30 species in 23 genera were included in the Bayesian and maximum likelihood analyses. The subfamily Coeliadinae, Eudaminae, Pyrginae and Heteropterinae were recovered as a monophyletic clade with strong support. The subfamily Hesperiinae formed a clade, but support for monophyly was weak. Our results imply that the five subfamilies of Chinese Hesperiidae should be divided into: Coeliadinae, Eudaminae, Pyrginae, Heteropterinae and Hesperiinae. The relationships of the five subfamilies should be as follows: Coeliadinae + (Eudaminae + (Pyrginae + (Heteropterinae + Hesperiinae))).

Reconstruction of the phylogenetic relationship of organisms plays an essential role in better understanding their evolution and diversification^1^. Lepidoptera, as the second largest order of insects with more than 157,000 species, are of particular interest in systematic research^2^,^3^. The skipper butterfly (Hesperiidae) which include around 4000 species is one of the most diverse groups of butterflies^4^,^5^. Although Hesperidae has been well defined, historically there exists disagreement at the subfamily and tribe levels.

The higher-level classification of Hesperiidae was established in the late 19th century. Watson divided Hesperiidae into three subfamilies (Pyrrhopyginae, Hesperiinae and Pamphilinae) based on the morphological characteristics of 201 genera^6^. The family was further arranged into six subfamilies by Evans^7^. Evans placed 130 genera in 4 subfamilies and 13 generic groups (equivalent to the current tribes), which shaped the current system for higher-level classification and interrelationships of the Hesperiidae. Studies on more detailed morphological characteristics have further advanced our knowledge important for classification and construction of phylogenetic relationships of the members in Hesperiidae^8^,^9^,^10^. Chou^11^,^12^ proposed three families in Hesperioidea (Euschemonidae, Megathymidae and Hesperiidae) and added three subfamilies (Coediadinae, Pyrginae and Hesperiinae) of Hesperiidae identified in China.

With information from molecular systematics studies in the past two decades, Warren *et al.*^13^ proposed the recent classification of Hesperiidae, to include five subfamilies: Coeliadinae, Pyrginae, Heteropterinae, Trapezitinae and Hesperiinae. With combined molecular and morphological data, Warren *et al.*^14^ subsequently revised the classification of Hesperiidae to include seven subfamilies: Coeliadinae, Euschemoninae, Eudaminae, Pyrginae, Heteropterinae, Trapezitinae and Hesperiinae. Warren’s molecular phylogeny included approximately 200 genera, representing about 35% of the skipper genera in the world. The skipper butterflies distributed in the Palaearctic and Oriental fauna were only partially covered. Less than half of skipper butterfly genera distributed in China are not known. The family Hesperiidae contains approximately 370 described species in 83 genera in China^15^ and so far there has been no molecular study of the higher-level phylogeny of the Chinese skipper butterflies. Mitochondrial DNA (mt DNA) sequence has been widely used in phylogenetic studies of Lepidoptera^16^,^17^,^18^. In this study, we used DNA sequences from the mitochondrial cytochrome b (*Cytb*), the NADH dehydrogenase subunit 1 (*ND1*) and cytochrome oxidase I (*COI*) to analyze the phylogenetic relationships of the genera in Hesperiidae.

## Materials and methods

### Taxon sampling

The butterflies studied were either collected with aerial nets in the field or were specimens in the Entomological Museum of Northwest A&F University in China. The specimens sampled and their collection site are listed in Appendix 1. A total of 30 skipper butterfly species in 23 genera were used in this study. In addition, five outgroup species, *Papilio protenor*, *Troides helena*, *Sericinus montelus* (Papilionidae), *Eurema andersoni*, *Pontia edusa* (Pieridae), from the Papilionoidea, the putative sister clade to the Hesperioidea^19^, were collected and used in this study.

### DNA extraction, PCR amplification and DNA sequencing

Total genomic DNA was extracted from a pair of legs of an adult specimen either dried or preserved in 95% ethanol, using the phenol-chloroform extraction protocol^20^,^21^. The genomic DNA prepared was dissolved in a 50 μL TE buffer and stored in a freezer (−20 °C).

PCR reactions were prepared in 50 μL that included 5 μL 10 × reaction buffer, 2.5 mM Mg^2+^, 0.6 mM primers, 4 μL of DNA template, 0.25 mM dNTPs and 1.0 U *Taq* polymerase. For amplification of the fragment from *Cytb*, the PCR amplification was performed by an initial denaturation at 94 °C for 5 min, followed by 35 cycles of 1 min at 94 °C, 1 min at 50 °C and 2 min at 72 °C, and a final extension at 72 °C for 10 min. For amplification of the fragment from *ND1*, the PCR cycles included 0.5 min at 94 °C, 0.7 min at 49 °C and 2 min at 72 °C. For amplification of the fragment from *COI*, the PCR cycles included 0.5 min at 94 °C, 0.7 min at 50 °C and 0.5 min at 72 °C. All primers used in this study were listed in Table 1. The PCR products of the PCR reactions from individual specimens were examined by agarose gel electrophoresis to verify the specific amplification of the desired fragments and the PCR products were sequenced for strands by commercial service (GeneScript Biological Technology, Nanjing, China, and Aoke Biological Technology, Beijing, China).

### Phylogenetic analysis

The DNA sequences from the individuals were aligned using MAFFT v7.037^25^, and the parsimony informative sites, base frequencies and Kimura-2-parameter distances (K2P distance) were calculated using MEGA v5.05^26^. The alignment was evaluated by substitution saturation using DAMBE v5.3.74^27^,^28^. The combined sequence datasets of *Cytb*, *ND1* and *COI* were used to construct phylogenetic trees.

Phylogenetic analysis by maximum likelihood (ML) model was conducted using jModelTest v 2.1.4^29^ using the Akaike information criterion (AICc). The best-fitting model of nucleotide substitution was GTR + I + G for all genes, and the general ratchet analysis conditions were as following: Lset base = (0.3552 0.0751 0.0866), nst = 6, rmat = (2.7601 10.2085 4.5566 7.6424 44.6148), rates = gamma, shape = 0.4670, ncat = 4, pinvar = 0.3640. PAUP* v4.0b10^30^ was used to calculate the ML analyses with 1000 bootstraps. Bayesian inference (BI) analysis was run in MrBayes 3.1.2^31^ using the model generated in jModelTest. The partitioned analysis comprised two runs with four Markov chain Monte Carlo simulations (MCMC) each, with flat priors, dataset partitioned by one million generations, sampling every 100 generations with 25% of samples discarded as burn-in.

## Results

### Sequence characterization

Alignment of the combined PCR fragment sequences from *Cytb*, *ND1* and *COI* showed that in the 1458 bp combined DNA sequences there were 717 variable sites and 568 parsimony-informative characters. The base composition of the fragments showed a strong bias of A + T (Table 2) as is commonly found in insect mitochondrial genomes^22^. The results of the substitution saturation test showed that the index of substitution saturation (*Iss*) was significantly lower than the critical value of the index of substitution saturation (*Iss.c*).

### Genetic distances

Calculation of the K2P distances between different species showed that they ranged from 0.1% (*Lobocla bifasciata*/*Lobocla liliana*) to 27.8% (*Eurema andersoni*/*Pontia edusa*) with an average genetic distance of 17.2%. The mean out- and in-group distance was 19.6% with a range of a minimal value of 13.8% (*Sericinus montelus*/*Choaspes hemixantha*) to maximal values of 25.2% (*Eurema andersoni*/*Daimio tethys*; *Eurema andersoni*/*Carterocephalus argyrostigma*). The mean in-group distance was 16.2% with a minimal value of 0.1% (*Lobocla bifasciata*/*Lobocla liliana*) and a maximum value of 22.4% (*Carterocephalus urasimataro*/*Satarupa nymphalis*). The mean distance was 14.6% (max. 18.8%) in the subfamily Hesperiinae and 15% (max. 17.8%) in Pyrginae.

### Phylogenetic relationships

**Figure 1:**
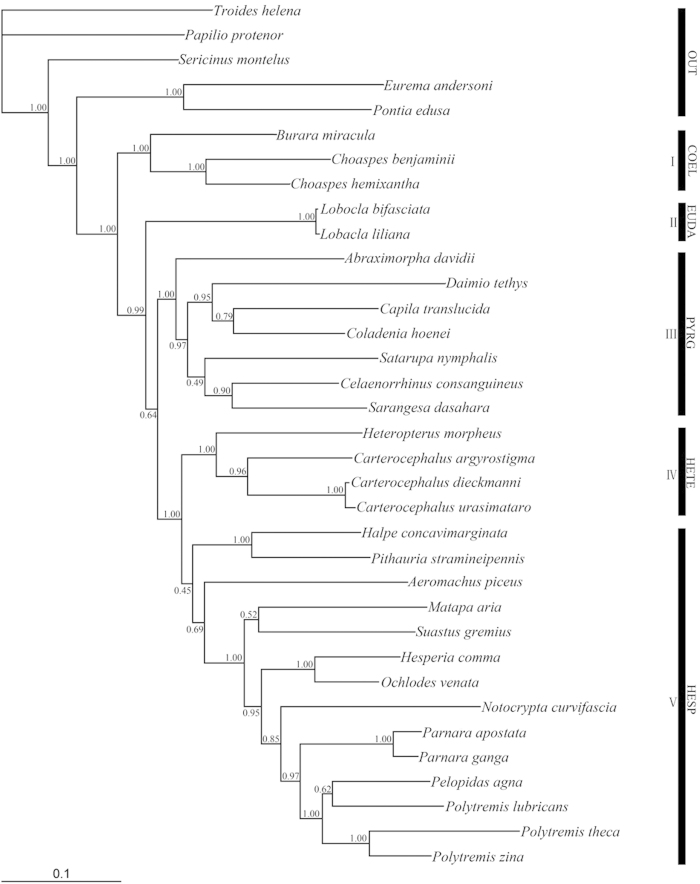
BI tree based on combined data of *Cytb, ND1* and *COI* partial gene sequences. Values above the branches indicate clade posterior probabilities. OUT, Outgroup; COEL, Coeliadinae; EUDA, Eudaminae; PYRG, Pyrginae; HETE, Heteropterinae; HESP, Hesperiinae.

The phylogenetic trees generated from the DNA sequence dataset by BI and ML methods trees are nearly identical in major clades and patterns of branching recovered. In BI analysis (Fig. 1), five of seven currently subfamilies of Hesperiidae were recovered as monophyletic clades with the following relationships: Coeliadinae + (Eudaminae + (Pyrginae + (Heteropterinae + Hesperiinae))).

The subfamily Coeliadinae (Clade I) was recovered as a monophyletic clade with strong support, although there were only three taxa (*Burara miracula*, *Choaspes benjaminii* and *Choaspes hemixantha*) included, and was placed in the basal position as the sister to the rest of the clades of the Hesperiidae. Although only two taxa within geneus *Lobocla* were included in our analysis from Eudaminae, its monophyly (Clade II) received strong support. The seven genera from Pyrginae formed a clade (Clade III) also with strong support. Furthermore, this clade split into three subclades: *Abraximorpha* + ((*Daimio* + (*Capila* + *Coladenia*)) + (*Satarupa* + (*Celaenorrhinus* + *Sarangesa*))).

The subfamily Heteropterinae (Clade IV) was monophyletic and strongly supported, although only two genera, *Heteropterus* and *Carterocephalus,* from this group were included in our analysis. In this clade, the geneus *Carterocephalus* included *C. argyrostigma*, *C. dieckmanni* and *C. urasimataro*, which were also recovered as a monophyletic group with strong support. As sister to Heteropterinae, the eleven genera from the Hesperiinae (*Halpe*, *Pithauria*, *Aeromachus*, *Matapa*, *Suastus*, *Hesperia*, *Ochlodes*, *Notocrypta*, *Parnara*, *Pelopidas* and *Polytremis*) appeared to form a clade (Clade V), but support for their monophyly is weak (<0.50). Within the Hesperiinae, the monophyly of Baorini (*Parnara*, *Pelopidas* and *Polytremis*), Ancistroidini (*Notocrypta*), Hesperiini (*Hesperia*, *Ochlodes*) and Isoteinonini (*Matapa*, *Suastus*) were recovered with strong or moderate support. However the Aeromacini (*Halpe*, *Pithauria*, *Aeromachus*) were not recovered as a monophyletic group.

**Figure 2:**
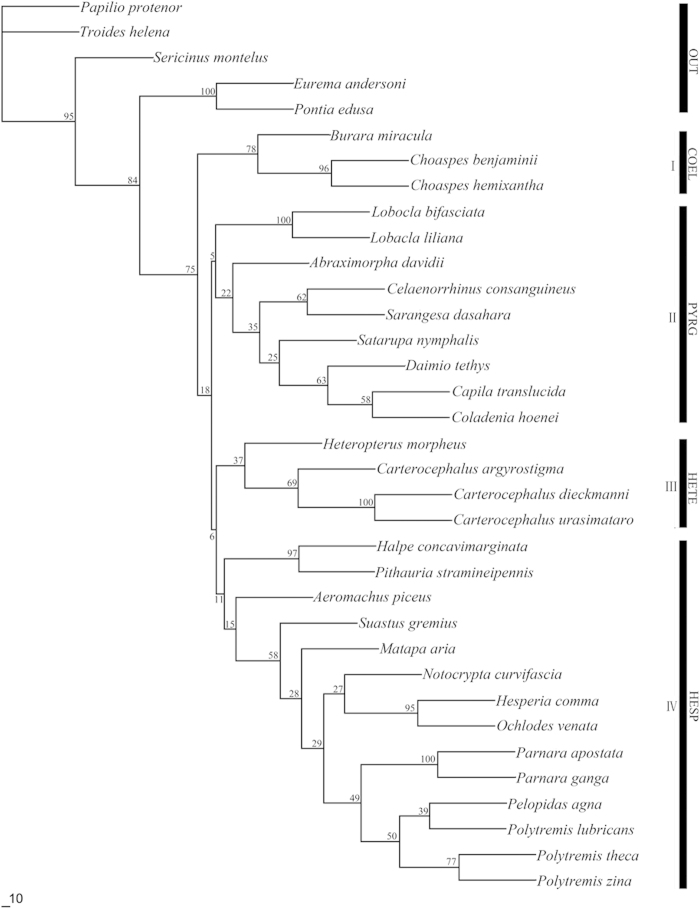
ML tree based on combined data of *Cytb, ND1* and *COI* partial gene sequences. Values above the branches indicate clade bootstrap support. OUT, Outgroup; COEL, Coeliadinae; PYRG, Pyrginae; HETE, Heteropterinae; HESP, Hesperiinae.

The phylogenetic tree by ML analysis showed that four major clades of Hesperiidae were recovered, although the relationships between some nodes were not strongly supported (<50) (Fig. 2). Compared with the topology of the tree by BI method, the major difference in the tree by ML is that the genus *Lobocla* was placed into the Pyrginae group (Clade II in Fig. 2), but the support was weak.

## Discussion

In the family Hesperiidae, Coeliadinae with morphological synapomorphies is relatively unique and easy to be distinguished from the remaining subfamilies^5^,^7^. The five genera (*Bibasis*, *Burara*, *Hasora*, *Badamia*, *Choaspes*) in this subfamily are distributed in China. In this phylogenetic study with two of these genera, the monophyly of Coeliadinae and its status as the sister of the rest of the Hesperiidae were confirmed, which is consistent with previous studies based on morphological and molecular data^13^,^14^,^32^,^33^. The morphological synapomorphy for Coeliadinae is the 3rd segment of labial palpi which is long, slender, cylindrical or awl-like^14^. Larvae generally feed on the plants of the class Dicotyledonopsida in China^15^.

The genus *Lobocla* was placed in the *Celaenorrhinus* group by Evans^7^; while, Warren *et al.*^13^ assigned it to the tribe Eudamini, which was then promoted to the subfamily of Eudaminae^14^. The result from the ML analysis of the mitochondrial DNA sequences in this study placed *Lobocla* in the subfamily of Pyrginae with weak support (Fig. 2). However, *Lobocla* became separated from the Pyrginae and formed an independent clade by BI analysis (Fig. 1). Given the higher value of posterior probabilities, we support the status of Eudaminae and that *Lobocla* as the only genus of Eudaminae occurring in the Oriental, Neotropical and Nearctic regions.

The subfamily Pyrginae has long been treated as a paraphyletic group^5^,^7^,^13^,^32^. Warren *et al.*^14^ recovered the monophyly of Pyrginae with moderate support. Pyrginae has been divided into seven tribes (Pyrrhopygini, Achlyodini, Tagiadini, Celaenorrhinini, Carcharodini, Erynnini and Pyrgini), but no morphological synapomorphies have been known for the subfamily. In this study, seven genera were included in the analysis and they appeared to form a monophyletic group with moderate support. However, the status of many other tribes and genera and their relationships within Pyrginae (e.g., *Caprona*, *Mooreana*, *Muschampia* in China) remain unknown. Additional taxa with additional molecular makers will be needed to elucidate their phylogenetic positions at the level of tribe and genera. Morphological characters for this subfamily are the 3rd segment of labial palpi which is short and stout, and that the larvae generally feed on plants of the class Dicotyledonopsida in China^15^.

The monophyly of the subfamily Heteropterinae was recovered with strong support (PP = 1.00) in the BI analysis (Fig. 1), which is consistent with the Warren *et al.*^14^ study. Heteropterinae is grouped under two tribes (Heteropterini and Carterocephalini), but morphological synapomorphies could be difficult to identify. Morphological characters for Heteropterinae are that the abdomens are distinctly elongated, longer than the length of the hindwing dorsum. Female bursa copulatrix has an appendix bursa. Larvae feed on plants of the class Monocotyledonopsida in China^15^. Within this subfamily, our results indicate that the genus *Carterocephalus* is a monophyletic clade in both analyses (PP = 0.96, BS = 69). However, the taxonomic status of this group remains to be resolved.

The subfamily Hesperiinae, as the largest subfamily of Hesperiidae, has long been a controversial subfamily in the Hesperiidae. The monophyly Hesperiinae has been reasserted^13^,^14^ and is also supported by the analysis in this study. Evans^7^ split the subfamily Hesperiinae into eight groups, and Inoué and Kawazoé^34^ reviewed Evans’s system, i.e., defining the *Halpe* group to include Evans’s *Astictopterus* group except for the genus *Astictopterus*. Chou^11^,^12^ divided the Chinese Hesperiinae into ten tribes based on Evans’s classification system. Warren *et al.*^14^ reviewed and recognized eight tribes of Hesperiinae (Aeromachini, Baorini, Taractrocerini, Thymelicini, Calpodini, Anthoptini, Moncini, Hesperiini). The results form this study support the monophyly of Baorini and Hesperiini. However, the classification status of other tribes has yet to be established with more taxa to be added to the phylogenetic analysis. The morphological character for Hesperiinae is the terminal part of lower margin of discal cell in hindwing which is oblique upwards. Larvae of this subfamily generally feed on plants of the class Monocotyledonopsida in China^15^.

In this comprehensive phylogenetic analysis of Hesperiidae members from China at subfamily-level with 30 species in 23 genera, the monophyly of this family was demonstrated with strong support. This result is in agreement with the previous reports by Wahlberg *et al.*^19^ and Warren *et al.*^13^,^14^, although higher level phylogenetic relationships remain challenging to decipher in Lepidoptera^3^. With strong posterior probability values, the results from BI analysis (Fig. 1) imply that the five subfamilies of Chinese Hesperiidae are under Coeliadinae, Eudaminae, Pyrginae, Heteropterinae and Hesperiinae. The relationships of the five subfamilies are Coeliadinae + (Eudaminae + (Pyrginae + (Heteropterinae + Hesperiinae))).

## Additional Information

**How to cite this article**: Xiangqun, Y. *et al.* Phylogenetic relationships of subfamilies in the family Hesperiidae (Lepidoptera: Hesperioidea) from China. *Sci. Rep.* **5**, 11140; doi: 10.1038/srep11140 (2015).

## Supplementary Material

Supplementary Information

## Figures and Tables

**Table 1:** Primers for PCR amplification ofgenes used in this study.

Gene	**Primer name**	**Sequence (5′-3′)**	**references**
*Cytb*	CB-J-10933	TATGTACTACCATGAGGACAAATATA	Simon *et al.*^22^
	CB-N-11367	ATTACACCTCCTAATTTATTAGGAAT	
*ND1*	3264-J-12095	ATCAAAAGGAGCTCGATTAGTTTC	Aubert *et al.*^23^
	1957-N-12567	CGTAAAGTCCTAGGTTATATTCAGATTCG	
*COI*	LCO1490-J-1514	GGTCAACAAATCATAAAGATATTGG	Folmer *et al.*^24^
	HCO2198-N-2175	TAAACTTCAGGGTGACCAAAAAATCA	

**Table 2:** Summary of number of taxa and characters for the three gene regions.

**Gene region**	**Number of sequences**	**Alignment length**	**A (%)**	**T (%)**	**C (%)**	**G (%)**	**Variable sites**	**Parsimony informative sites**	***Iss* values**	***Iss.c* values**
*Cytb*	34	408	30.6	43.5	16.6	9.4	215	182	0.373	0.692
*ND1*	34	446	30.7	49.0	8.3	12.0	260	193	0.400	0.696
*COI*	30	604	30.7	39.7	16.0	13.7	242	193	0.373	0.692
*Combined*	35	1458	30.6	43.8	13.7	11.8	717	568		
