# Supplementary material for: Phylogenetic relationships of subfamilies in the family Hesperiidae (Lepidoptera: Hesperioidea) from China
Source: Sci Rep. 2015 Jun 10;5:11140. doi: 10.1038/srep11140 (PMC4461911; doi:10.1038/srep11140)
Supplement: Supplementary Information [file srep11140-s1.pdf]

Phylogenetic relationships of subfamilies in the family Hesperiidae (Lepidoptera: Hesperioidea) from China

Yuan Xiangqun, Gao Ke, Yuan Feng, Wang Ping, Zhang Yalin

Appendix 1. List of Hesperiidae and outgroups used in this study, with collection localities and GenBank accession numbers

Subfamily	Species	Locality	GenBank accession no.		
			Cytb	*ND1*	*COI*
Coeliadinae	*Burara miracula*	Fujian, Wuyi Mountain	KM895429	KM895451	KM895473
	Choaspes benjaminii	Jiangxi, Ruijin	KM895435	EU783937	KM895476
	Choaspes hemixantha	Jiangxi, Suichuan	KM895436	KM895457	KM895477
Eudaminae	*Lobocla bifasciata*	Yunnan, Zhongdian	KM895441	KM895462	GU372595
	Lobocla liliana	Yunnan, Zhongdian	KM895442	KM895463	—
Pyrginae	*Capila translucida*	Sichuan, Emei Mountain	KM895430	KM895452	KM895474
	Celaenorrhinus consanguineus	Yunnan, Zhongdian	KM895434	KM895456	KM895475
	Abraximorpha davidii	Hainan, Changjiang	KM895427	KM895449	KM895471

	Daimio tethys	Shaanxi, Tongchuan	KM895438	KM895459	KM895479
	Coladenia hoenei	Shaanxi, Feng County	KM895437	KM895458	KM895478
	Satarupa nymphalis	Gansu, Hui County	KM895446	—	GU372591
	Sarangesa dasahara	Yunnan, Jinghong	—	KM895468	HQ962347
Heteropterinae	*Heteropterus morpheus*	Jilin, Changbai Mountain	KM895440	KM895461	JX445978
	Carterocephalus argyrostigma	Gansu, Xishu	KM895431	KM895453	—
	Carterocephalus dieckmanni	Yunnan, Zhongdian	KM895432	KM895454	—
	Cartericephalus urasimataro	Shaanxi, Taibai County	KM895433	KM895455	—
Hesperiinae	*Halpe concavimarginata*	Fujian, Wuyi Mountain	KM895439	KM895460	KM895480
	Pithauria stramineipennis	Yunnan, Menglun	KM895445	KM895467	KM895485
	Aeromachus piceus	Fujian, Wuyi Mountain	KM895428	KM895450	KM895472
	Matapa aria	Fujian, Wuyi Mountain	KM895443	KM895464	KM895481
	Suastus gremius	Fujian, Fuzhou	KM895447	KM895469	—
	Hesperia comma	Liaoning, Wuge Mountain	KJ775048	KJ786304	HM393208

	Ochlodes venata	From GenBank	JQ924458	KM895466	HM391838
	Notocrypta curvifascia	Fujian, Wuyi Mountain	KM895444	KM895465	KM895482
	Parnara apostata	Yunnan, Menglun	KJ775037	KJ786293	KM895483
	Parnara ganga	Fujian, Wuyi Mountain	KJ775035	KJ786291	KM895484
	Pelopidas agna	Yunnan, Menglun	KJ775043	KJ786299	KF387988
	Polytremis lubricans	Hainan, Baisha	KJ775038	KJ786295	KM895486
	Polytremis theca	Fujian, Wuyi Mountain	KJ775040	KJ786297	KC684413
	Polytremis zina	Zhejiang, Wuyanling	KJ775039	KJ786296	KC684395
Papilionidae	*Troides helena*	Shaanxi, Yangling	KM895448	KM895470	KM895487
	Papilio protenor	Yunnan, Jinping	KM669332	KM669387	HM246460
	Sericinus montelus	Shaanxi, Taibai County	KM669333	KM669388	KF723528
Pieridae	*Eurema andersoni*	Shaanxi, Taibai County	KM669291	KM669343	HM246460
	Pontia edusa	Yunnan, Xishuangbanna	KM669318	KM669371	KC462876

—, data not available.
